# Microbes go nano

**DOI:** 10.1111/1751-7915.12434

**Published:** 2016-11-14

**Authors:** Jesús M. Sanz, Beatriz Maestro

**Affiliations:** ^1^ Miguel Hernandez UniversityElcheSpain

## Abstract

Walk on the small side. Nanotechnology meets Microbiology thanks to the high versatility of synthetic routes in microorganisms, leading to the production of nanoparticles of biotechnological and biomedical interest.

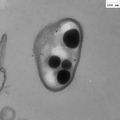

It is no secret that Nanotechnology, despite its name, is continuously growing up. Synthetic nanostructures have demonstrated an invaluable potential in biomaterial, electronic and biomedical sciences, with multiple applications and with a wide open horizon. Concomitantly, the links of Nanotechnology with Microbiology are strengthening in the last years. A PubMed search including the string *Microbiology and Nanotechnology or Nanobiotechnology or Nanoparticles* reveals an exponential increase in citations from 3 in 1998 to 779 in 2015. Contributing to this increment, the use of microbes as nanoparticle (NP) factories is continuously revealing its potential. Usual chemical synthesis of NPs is hampered by the high cost, complexity and toxicity of the process steps. Therefore, the possibility of employing microbes to biosynthetize these compounds is attracting much attention, as these eco‐friendlier systems allow an easier downstream purification process – and indeed, quite promising results are showing up.

Most microbe‐produced NPs are of metallic nature. Metal NP deposition is caused by reduction of a metal salt using the redox machinery in the cell. Cations that are found in the microbial environment taking part in metabolic processes are reduced and precipitated by some bacteria as NP if their concentration exceeds a limit, so that NP formation is actually a protective mechanism (Tanzil *et al*., 2016). Several microorganisms such as *Geobacter sulfurreducens and Shewanella oneidensis* are able to generate these structures. Moreover, a variety of molecular synthesis mechanisms, either inside or outside the cell, have been described, and also both size and morphology of NPs depend on environmental conditions such as pH and medium composition. Therefore, much more investigation is on the way to fully explain the processes involved, which is necessary to increase and fine‐tune a rational and homogeneous NP production. In this sense, use of biofilms might be advantageous in terms of high biomass concentration and better scaling‐up, besides being less prone to contamination than liquid cultures (Tanzil *et al*., 2016).

Magnetotactic bacteria have been long known as producers of natural magnetic NPs or magnetosomes (reviewed by Jacob and Suthindhiran, 2016). These membrane‐coated metallic bodies help the microorganism, mainly belonging to the *Magnetospirillum* genus, to be guided along the Earth magnetic field. One strong advantage of magnetosomes over chemically synthetized magnetic NPs is their homogeneity in terms of size and shape. This has been employed in a high number of applications in biomedicine such as drug delivery and as imaging probes, as well as bioremediation (Jacob and Suthindhiran, 2016). Nevertheless, the most important drawback resides in the difficult culturing of magnetotactic bacteria, which is highly dependent on small variations on carbon, iron and oxygen sources, and needing redox gradient conditions that are hard to reproduce in the laboratory (and not to mention an industrial bioreactor). Therefore, efficient scaling‐up is the major challenge to be solved for the successful biotechnological production of these interesting NPs. On the other hand, more biocompatibility studies must be carried out to ensure the safe application of magnetosomes in biomedicine.

An interesting approach is that represented by works such as of Fernández‐Llamosas *et al*. (2016). The authors employed the ability of *Azoarcus* sp. CIB to reduce selenite to selenium NPs (Fig. 1). Since this strain degrades different toxic aromatic hydrocarbons, the possibility of NP production coupled to environment bioremediation foresees appealing applications.

**Figure 1:**
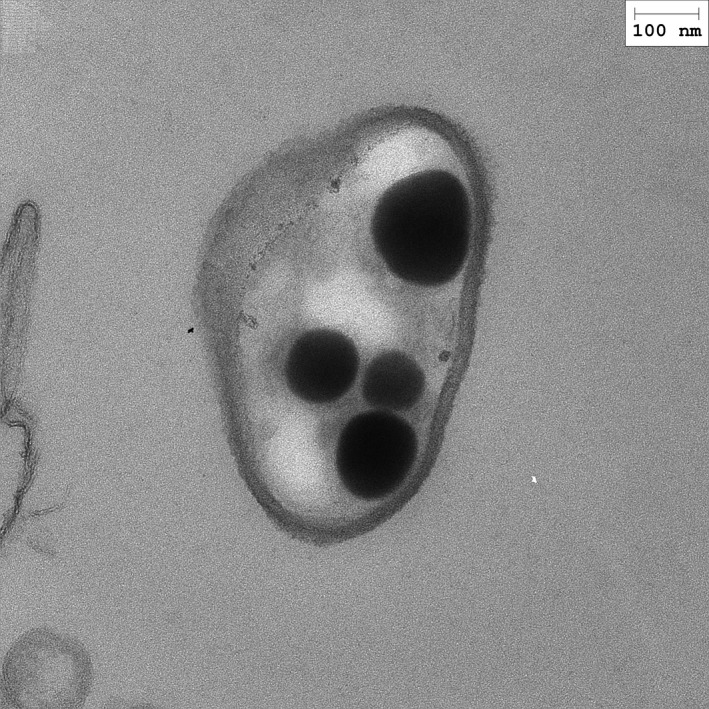
Accumulation of selenium nanoparticles inside *Azoarcus* sp. CIB cells (courtesy of Dr. M. Carmona).

Indeed, not all microbial‐synthetized NPs are metallic in nature. The polyhydroxyalkanoate (PHA) industry is well known, and constitutes one of the most active fields of research in Biotechnology, as these natural biopolymers have the potential of successfully compete with oil‐derived plastics in some applications. Accordingly, biosynthesis of PHA nanobeads is the subject of an intense research (Dinjaski and Prieto, 2015) since downstream processing of PHA intracellular granules involves less costly processes than *in vitro* procedures. Current efforts to boost PHA NPs commercialization are focused on investigating methods to control the structure and size of the nanobeads, as well as on further decreasing production costs and on finding high added‐value applications.

Will we soon contemplate the burst of NP fabrication by microbial cell factories? Very probably – but some more research still needs to be carried out. A better knowledge of NP biosynthetic routes will help to design procedures for a tighter control of NP size, homogeneity and stabilization. On the other hand, improvement of control of microbial growth conditions will ensure an efficient scaling‐up from the laboratory bench to industrial reactors, which is key for mass production. Finally, biocompatibility issues must be addressed as a prerequisite for a fruitful biomedical application. In so far as these aspects are optimized, we will witness the progressive implementation of microbial‐based NP biotechnology.
